# Adjusting Daily Inpatient Bed Allocation to Smooth Emergency Department Occupancy Variation

**DOI:** 10.3390/healthcare8020078

**Published:** 2020-03-28

**Authors:** Jeffrey Che-Hung Tsai, Shao-Jen Weng, Shih-Chia Liu, Yao-Te Tsai, Donald F. Gotcher, Chih-Hao Chen, Chun-An Chou, Seung-Hwan Kim

**Affiliations:** 1 Department of Emergency Medicine, Taichung Veterans General Hospital, Puli Brach, Nantou 54552, Taiwan; erdr2181@gmail.com; 2 Department of Industrial Engineering and Enterprise Information, Tunghai University, Taichung 40799, Taiwan; liushihchia@gmail.com (S.-C.L.); p8311011@gmail.com (C.-H.C.); 3 Healthcare Systems Consortium, Tunghai University, Taichung 40704, Taiwan; 4 Department of International Business, Feng Chia University, Taichung 40723, Taiwan; yaottsai@fcu.edu.tw; 5 Department of International Business, Tunghai University, Taichung 40704, Taiwan; dongotcher@yahoo.com; 6 Department of Mechanical and Industrial Engineering, Northeastern University, Boston, MA, 02115, USA; ch.chou@northeastern.edu; 7 Department of Business Administration, Ajou University, Suwon 443-749, Korea; seunk@ajou.ac.kr

**Keywords:** overcrowding, inpatient bed allocation, emergency department operations, discrete-event simulation

## Abstract

Study Objective: Overcrowding in emergency departments (ED) is an increasingly common problem in Taiwanese hospitals, and strategies to improve efficiency are in demand. We propose a bed resource allocation strategy to overcome the overcrowding problem. Method: We investigated ED occupancy using discrete-event simulation and evaluated the effects of suppressing day-to-day variations in ED occupancy by adjusting the number of empty beds per day. Administrative data recorded at the ED of Taichung Veterans General Hospital (TCVGH) in Taiwan with 1500 beds and an annual ED volume of 66,000 visits were analyzed. Key indices of ED quality in the analysis were the length of stay and the time in waiting for outward transfers to in-patient beds. The model is able to analyze and compare several scenarios for finding a feasible allocation strategy. Results: We compared several scenarios, and the results showed that by reducing the allocated beds for the ED by 20% on weekdays, the variance of daily ED occupancy was reduced by 36.25% (i.e., the percentage of reduction in standard deviation). Conclusions: This new allocation strategy was able to both reduce the average ED occupancy and maintain the ED quality indices.

## 1. Introduction

Overcrowding in emergency departments (ED) has become an increasingly significant public health problem [1,2,3,4], which leads to detrimental effects on patient care, ED efficiency, and patient and provider satisfaction [5,6,7]. Some investigators reported that ED overcrowding causes treatment delays, higher mortality rates, prolonged inpatient lengths of stay (LOS), and hospital readmissions [8,9]. The increase in the number of emergency visits in Taiwan has been a growing trend in recent years. Annual emergency visits increased by 20% from 1999 to 2013 [10]. ED admissions in Taiwan have also increased by 38% from 1998 to 2011 [11]. This rise in intake results in overcrowding and longer wait times. In EDs at 4 out of 19 medical centers, more than five percent of their patients were held for periods of more than 48 h (2014 statistics). In other words, these patients had to stay in the hospital for more than 48 h awaiting a physician’s admission decision. In addition, of the ED patients who did get admitted to the hospital, 9.3% of these patients were held for more than 48 h without assigning ward beds in some of the medical centers, and these figures were even higher than 10% in eight out of 19 medical centers (with one at an extremely high 26.1% figure) [12]. Hospital bed availability and hospital occupancy are determined by the daily bed allocation of five major types of patients competing for empty beds. These include those with elective surgery, from the ED, invasive examination, medical examination, and transfers from the intensive care unit (ICU). Therefore, the situation where these patients have to contest for limited medical resources leads to inefficient operations and is one of the reasons for admission congestion.

It is critical at the hospital management level to provide support in easing admission congestion; such support is often met with financial barriers in the hospital. In the admission of elective surgical patients, they are usually prioritized into wards. Such prioritization is linked to the way reimbursements are delivered according to the national medical insurance regulations. For these patients, the reimbursement is higher, and their lengths of stay are lower than those patients from the ED. In addition to maximizing medical revenues, hospitals desire to maximize full capacity, and the ED is one area where patients can be admitted despite its being crowded [13].

Overcrowding of EDs is defined as the situation in which ED functions are impeded primarily because of the excessive number of patients waiting to be examined, assessed and treated, or in waiting for departure compared to the physical or staffing capacity of the ED [14]. Overcrowding in the ED can occur under the following situations: a large volume of patients waiting to be seen (input), long delays in assessing or treating patients already in the ED (throughput), or impediments to patients leaving the ED after treatment (output) [8,9]. Overcrowding is not simply generated by ED flows; it is also the result of admission congestion [15]. Access blocks refer to the situation where incoming patients at the ED requiring inpatient care are unable to gain access to the appropriate hospital beds within a reasonable time frame [7,16,17,18]. In theory, efficient hospital operation should help in speeding up bed access. 

The ED overcrowding problem can be solved in the following three ways: (a) limiting emergency visits (input); (b) increasing the number of discharged/transferred patients and the admission capacity (output), and (c) improving the efficiency of ED flow (throughput) [8]. Much of the existing literature has proposed solving the ED overcrowding problem with the following measures: fast-tracking, reclassification of patient flow, flow improvement of examinations, the participation of physicians in triage, ED flow improvement, simulation-based improvement, and lean management [13,19,20,21,22,23,24,25,26]. Experts recommend that the single most important factor for ED diversion is the daily variability in the elective surgical caseload [2]. When weekly or daily peaks and valleys in demand for admission are predictable, hospitals can adopt “smoothing” strategies to even admissions across all weekdays [27]. In addition, the most important indicators documenting ED overcrowding were the percentage of the ED occupied by inpatients, the total number of ED patients, and the total time stayed in the ED [21]. ED capacity varies across hospitals, therefore no fixed number can be used to indicate the condition of overcrowding, which happens whenever patient volumes surpass the ED capacity of hospitals. 

In one study, the relationship between hospital occupancy and the number of ED admissions was analyzed in time series against lengths of daily stay. Three factors were found to be independently associated with lengths of daily stay: (a) The number of elective surgical admissions. These admissions are for patients with the surgery scheduled in advance; (b) the number of ED admissions. These admissions account for unplanned or urgent patients; and (c) hospital occupancy. This factor represents the occupancy rate of a hospital. The mean length of a daily stay is 0.21 min longer for every additional elective surgical admission, 2.2 min longer for every additional admission, and 4.1 min longer for every 5% increase in hospital occupancy [28]. Thus, we hypothesize that an increase in hospital occupancy is associated with the length of ED stay for admitted patients, and an increase in the available hospital beds will reduce ED overcrowding [29,30].

In recent years, simulation-based approaches have been used to answer “what if” questions, in order to achieve more efficient healthcare services [31]. Due to their complex and stochastic nature, simulation is used as a tool to analyze critical parts of healthcare systems such as facility design (e.g., ED, operating room), staff planning/scheduling, occupancy flows, and bed capacity management [26,32,33,34,35]. Using simulation, a study was done on an ED in a British hospital, with the objective of determining the impact of key resources (such as waiting times, waiting lines, and throughput) [31].

Simulation is useful in exploring problems of ED overcrowding for the following reasons: overcrowding is a complex phenomenon that can be reflected in terms of different measures, such as numbers of waiting patients, boarding patients, and occupied beds. In addition, simulation can output a detailed list of upcoming patients predicted to stay in the ED. Based on this information, the forecasts for outcome measures can be derived [36]. Thus, simulation is not only about data collection and output analysis, but it can also identify complexity of the system and help in developing a valid model that is useful for optimizing decision-making [20].

In this study, we used simulation specifically to assess temporal variations in ED bed occupancy and to evaluate the effects of leveling variation in ED occupancy by adjusting the inpatient bed availability each day. We aimed to propose feasible solutions and obtain an optimized bed allocation strategy to reduce ED overcrowding.

## 2. Materials and Methods

### 2.1. Model Development

**Figure 1:**
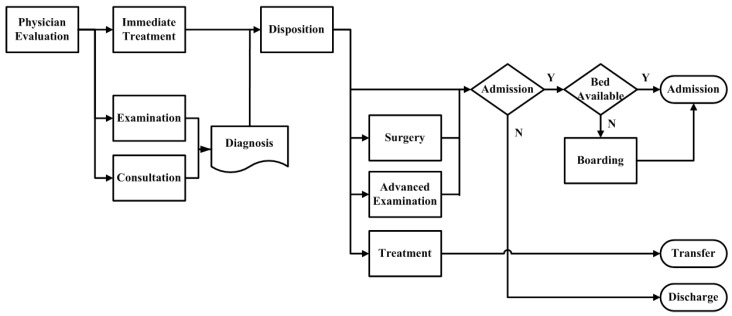
Emergency department operation flow chart.

Our model was constructed to analyze administrative data recorded in the ED of Taichung Veterans General Hospital (TCVGH), which is a medical center located in central Taiwan. It has 1500 beds with an annual ED volume of 66,000 visits. The ED has been over-crowded for many years. In 2014, 17.4% of ED patients stayed there for more than 24 h, and 7.7% for more than 48 h. Before getting the admission disposition (decision) of the physician, these patients would not get admitted to the hospital nor get discharged. In other words, they are one of the reasons for the ED being overcrowded. For those patients who were admitted, over 1/3 (35.2%) of them were held in the ED for more than 24 h, and 15.0% of them were held for more than 48 h. The data were collected during the period from 1 November 2017 to 31 July 2018. Key data included the patient waiting times at each station in the ED, the number of emergency beds, and the surgical bed resources. Figure 1 shows the ED workflow diagram. After evaluation by the physician, ED patients get immediate treatment, initial examination, and/or consultation. Next, the physician diagnoses and decides if these patients should be assigned for surgery, advanced examination, or treatment. Patients who need to get surgery or advanced examination are admitted. These admitted ED patients then wait for assignment of available ward beds. Other patients, after appropriate examinations and treatments, are either transferred to another hospital or discharged from the ED. For privacy protection, the study was approved by the Institutional Review Board (IRB) of Taichung Veterans General Hospital (TCVGH No: CE17205A).

In this study, we developed through the SIMUL8^®^ software (SIMUL8 Corporation, Boston, MA, USA), a discrete-event simulation model based on the ED workflow diagram, and used it to test the feasibility of reducing overcrowding. Previous simulation-based studies have predicted the number of available ICU beds and evaluated the cost of patient transportation to hospitals [37,38]. Simulation has also been applied in patient queuing. For example, a simulation model was used to analyze hospital emergency care flows with the aim of improving the flows, resource allocation, and patient waiting times [39]. Other relevant research works are also reported [40,41]. 

We built a queuing model based on the speed of services provided to patients with different triage scale evaluations (Taiwan Triage and Acuity Scale). There are five acuity levels, which are level 1 (resuscitation), level 2 (emergent), level 3 (urgent), level 4 (less urgent), and level 5 (not urgent). For the process of ED operations, queuing theory is able to provide a better understanding of the mechanism [42]. There are several queuing models such as one server (M/M/1), multiserver (M/M/C), multiclass, multiserver (MCMS), which usually consist of four major components, patient source, queue, queuing rules, and service organization [43,44]. In our system, a set of M distinct stations, indexed by j = 1,…,M, serve K patient classes with index I = 1,…,K. These patients are served by a subset of stations. At time t = 0, a number of customers in different classes (N) come into the system (arrival rate = λ) waiting for service (service rate = μ). In general, patients are first-come, first-served, but priority will be given to patients with higher triage scale levels, in our case, levels 1 and 2. In addition, we analyzed the supply and demand of ED beds on daily, weekly, and monthly basis, and included these components in our model. In particular, the model was proposed to determine the appropriate bed allocation between available ED beds and patient occupancy (total patients staying in the ED).

In our simulation model, several patient and hospital-related parameters were considered: arrival of medical patients to the hospital, arrival of surgical patients to the hospital, waiting time in queuing for examinations, waiting time for getting reports, triage scale determinations, waiting time in queuing for treatments, percentage of those getting examinations, percentage of those discharged, waiting time for transfers, and the percentage of those transferred. Raw patient data were used to fit the distribution of the parameter settings in the model. The model was designed to examine four major crowding indicators in the ED:

1. Proportion of patients held >24 h. This is the percentage of patients who stay in the ED for more than 24 h with no physician admission disposition.
2. Proportion of patients held >48 h. This indicates the percentage of patients who stay in the ED for more than 48 h with no physician admission disposition.
3. Proportion of admitted patients held >24 h. This represents the percentage of patients who get admitted to the hospital and wait for more than 24 h with no available bed assigned.
4. Proportion of admitted patients held >48 h. This is the percentage of patients who get admitted to the hospital and wait for more than 24 h with no available bed assigned.

### 2.2. Current ED Operations

**Figure 2:**
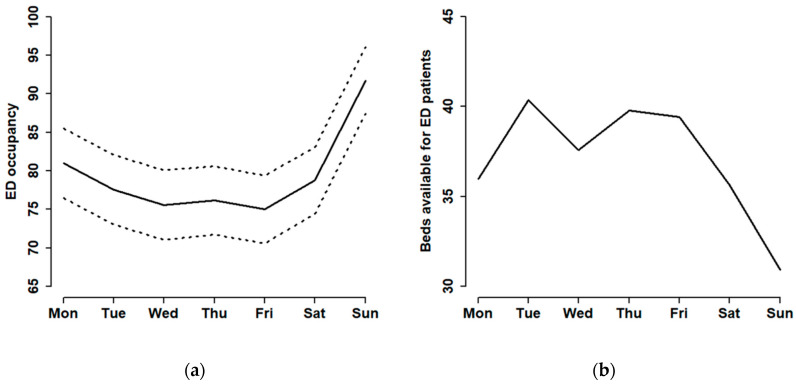
The mean and 95% CI of emergency department (ED) occupancy (**a**), and the mean of numbers of beds available for ED patients (**b**) shown on a daily basis throughout the week.

According to the ED operational data provided by the hospital, the average ED occupancy ranged between 75 and 92 percent, and it reached a peak number on Sundays. In addition, the approximate difference between the lower and upper bounds of the confidence interval is 10 (Figure 2). The data of available beds per day for ED patients showed the lowest number on Fridays. ED occupancy on other weekdays also showed lower numbers compared to weekends. This situation was opposite to the ED occupancy (Figure 2). The difference between the highest and lowest average numbers of available beds for ED patients from Monday to Sunday was only 9 beds. This is a common issue in the healthcare industry in Taiwan. While the variation between weekdays and weekends is huge, the number of available beds for ED patients is not adjusted effectively.

### 2.3. Analysis of the Strategy 

High variations ($\sigma^{2}$ is close to 20, at a 95% confidence interval of ED occupancy) might lead to poor allocation of human resources, and lower surge capacity for disaster response. The target of our study was to minimize the variance of ED occupancy by leveling the number of available acute care beds for ED patients. We used a simulation model to analyze different scenarios for ED bed allocation. The total number of allocated acute care beds for ED patients was kept constant on a weekly basis. In our scenarios, we tended to reallocate several ED beds from weekdays to weekends. We simulated the current operation in scenario 1 and compared it to five scenarios with different degrees of ED bed reduction from weekdays. For example, we reallocated five percent of weekday ED beds to weekends in scenario 2, 10 percent in scenario 3, 15% in scenario 4, 20% in scenario 5, and 30% in scenario 6. In scenario 2, the average available weekday ED beds were reduced from 32.49 to 30.93, while the available weekend ED beds increased from 28.07 to 31.97. The numbers of ED beds available under different scenarios are presented in Table 1.

## 3. Results

**Figure 3:**
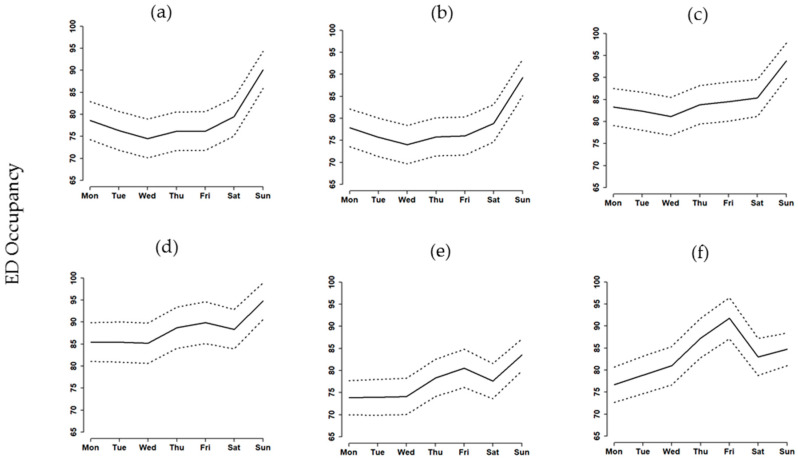
The mean and 95% CI of ED occupancy under different scenarios: (**a**) S1 (average), (**b**) S2 (−5%), (**c**) S3 (−10%), (**d**) S4 (−15%), (**e**) S5 (−20%), and (**f**) S6 (−30%).

Table 2 shows the simulated average ED occupancy and smoothing effects, which is the reduction in percentage of standard deviation (Std), based on the current operations, simulated operations, and the five scenarios we proposed. Results indicate that S1 reduces 9.62% of the smoothing effects from the current operations, while S2, S3, S4, and S5 reduced smoothing effects by 12.03%, 28.52%, 41.24%, 36.25%, and 11.65%, respectively. Although S3 and S4 had higher smoothing effects, the ED occupancies are higher than the current operations. Therefore, these two scenarios would not be considered as feasible strategies. S5 performed the best among all scenarios with a 36.25% reduction in Std and with a lower ED occupancy. We also note that reducing the number of ED ward beds on weekdays by 30% as in S6, resulted in poorer performance with a higher number for ED occupancy and a lower number for smoothing effects compared with S3, S4, and S5. S4 had the greatest smoothing effect compared to the weekly baseline, but S5 had an average ED occupancy lower than S4 (Figure 3). We also found that an excessive reduction in the number of weekday ED beds resulted in a surge of high ED occupancy on Fridays.

When the four ED overcrowding indicators were compared across scenarios, we found that a mild reduction in the number of ED beds on weekdays reduced the smoothing effects of ED occupancy. On the other hand, an excessive reduction in the number of ED beds caused negative effects on the ED crowding indicators. For example, the number of ED patients held >24 h was increased at reductions of 10% and 15% of ED beds. This indicator decreased at a reduction of 20%. However, a 30% ED bed reduction increased ED patients held >24 h. Similar trends of changes against extents of reduction were also found with other indicators. When we compared all the scenarios, scenario 5 is the only candidate with similar indicator performance compared to the current operations and highest smoothing effects. (See Table 3).

## 4. Discussion

The aim of our present study is to propose a bed allocation strategy for solving the overcrowding problem in the ED. To be more specific, the strategy is able to control a relatively flatter level of ED occupancy, or in essence, smoothing the variance of the ED occupancy. We used a simulation approach to develop different scenarios of ED bed allocation. Our study showed a reverse relationship existing between ED occupancy and the average allocated beds for ED patients across different days of a week. The average number of patients staying in the ED was between 75 and 92 per day, which climbed to a peak number on Sundays. Conversely, the average number of available beds for ED patients was lower on weekends. The key point flowing from the strategic analysis using our simulation mode is that we reduced the number of available ED beds on weekdays and shifted them to the weekends in order to get a better balance of bed allocation for ED patients. We found that if we reduced the allocated beds for the ED by 20% during weekdays, a significant improvement in the smoothing effect (36.25%) on daily ED occupancy can be achieved in the strategic analysis using our simulation model. This strategy also reduced the average ED occupancy and maintained the non-compromised quality indicators of ED overcrowding. The smoothing of ED occupancy rates can allow a predictable allocation of manpower and avoid work overload of ED health care providers. The strategy of maintaining a more constant total number of allocated beds for ED patients in our study can also maintain the current balance of competition between ED patients and non-ED patients for acute care beds. The results are the preservation of hospital revenues and the avoidance of financial barriers to support measures easing ED overcrowding. The hospital administration can, therefore, provide more beds for ED patients during weekends, and fewer beds for them on the following weekdays when ED volume is lower. 

We suggest that the findings should be implemented in select hospitals in an effort to solve the ED crowding problem. Consequently, manpower allocation will be more predictable, human resources can be provided more efficiently, and work overload of ED health care providers can be avoided. Adjusting schedules of elective surgeries among different specialty teams may further support this strategy of leveling inpatient beds allocation for ED patients.

## 5. Conclusions

ED overcrowding problem has been one of the serious issues globally. Not only the insufficient bed allocation would lead to ED overcrowding, but also reduces ED quality. The contribution of this study is that we provide an effective bed allocation strategy to ED for improving the resource utilization and reducing overcrowding. More specifically, the strategy is able to level the variance and balance the number of excess weekday ED beds and insufficient weekend beds so that the ED patients would gain better ED service quality.

## Figures and Tables

**Table 1:** Leveling numbers of acute care beds for ED patients.

Scenario	Weekdays	Weekends (Adjusted to Maintain the Same Total Number on a Weekly Basis)
S1	32.49 (average)	28.07
S2	30.93 (−5%)	31.97
S3	29.37 (−10%)	35.87
S4	27.80 (−15%)	39.78
S5	26.24 (−20%)	43.68
S6	23.12 (−30%)	51.48

**Table 2:** The ED occupancy between different scenarios.

Scenario	Mean	Std ^a^	Smoothing Effects ^b^
Original	79.40	5.82	baseline
S1 (average)	78.77	5.26	9.62%
S2 (−5%)	78.23	5.12	12.03%
S3 (−10%)	84.91	4.16	28.52%
S4 (−15%)	88.26	3.42	41.24%
S5 (−20%)	77.42	3.71	36.25%
S6 (−30%)	83.33	5.14	11.68%

^a^ Std: standard deviation; ^b^ The smoothing effect is the percentage of the reduction in standard deviation on ED occupancy in each of the scenarios compared with the baseline.

**Table 3:** ED overcrowding indicators in different scenarios.

Strategy	ED Patients Held >24 h	ED Patients Held >48 h	Admitted ED Patients Held >24 h	Admitted ED Patients Held >48 h
Original	8.44	4.86	30.12	17.32
S1 (average)	8.63	4.83	30.79	17.22
S2 (−5%)	8.58	4.79	30.60	17.08
S3 (−10%)	10.12	6.00	36.11	21.43
S4 (−15%)	10.40	6.18	37.15	22.07
S5 (−20%)	8.80	4.90	31.37	17.58
S6 (−30%)	10.07	5.41	35.90	19.31
